# Death and fire—the concept of necroinflammation

**DOI:** 10.1038/s41418-018-0218-0

**Published:** 2018-11-23

**Authors:** Andreas Linkermann

**Affiliations:** Division of Nephrology, Department of Internal Medicine III, University Hospital Carl Gustav Carus at the Technische Universität Dresden, Dresden, Germany

**Keywords:** Chronic inflammation, Cell death and immune response

Death may be either immunogenic or not, and fire (inflammation) (Fig. 1) potentiates the cellular damage, but also initiates organ regeneration. With close to 200,000,000,000 cells dying in our bodies each day by apoptosis, it is obvious that this pathway of regulated cell death does not cause severe inflammation. However, more recently discovered necrotic type cell death pathways, such as necroptosis, ferroptosis, and pyroptosis, inevitably release intracellular damage-associated molecular patterns (DAMPs). In an organism, this leads to access of tissue-resident immune cells directly to proimmunogenic intracellular factors. Clinically important examples in which necroinflammation is involved are antinuclear antibodies (ANAs) and anti-double-stranded DNA antibodies, which drive human autoimmune diseases.

**Fig. 1:**
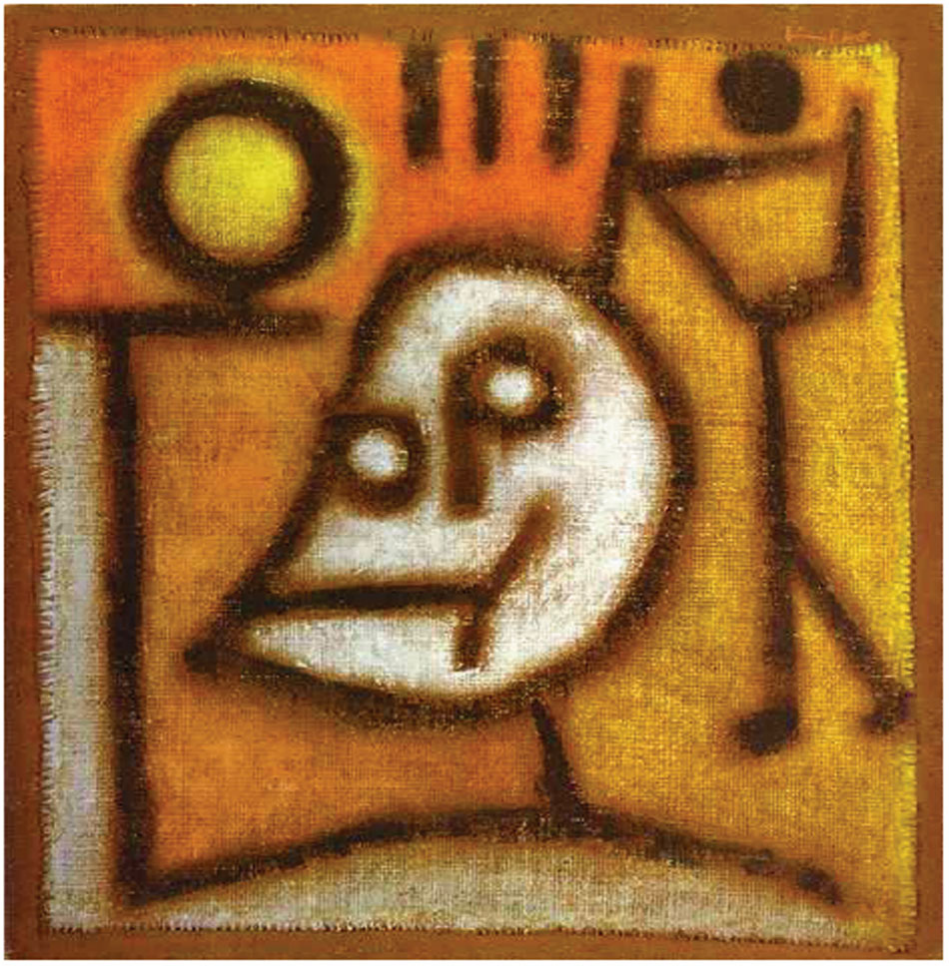
**Tod und Feuer, Paul Klee 1940 (Zentrum Paul Klee, Bern, Switzerland**, ***used with permission***). In 1940, the German expressionist Paul Klee painted “Tod und Feuer” (“Death and Fire”). Oil on distemper on jute. With himself passing away on June 29 of the same year, it is today considered one of his final masterpieces. Klee at this time suffered from an autoimmune disease known as scleroderma. The disease heavily affected his joints and hardly allowed painless painting. Scleroderma is associated with the development of autoantibodies, the most common ones being anti-nuclear antibodies, and represents a typical example of a degenerative disease driven by necroinflammation

Two paramount features are associated with necrotic cellular damage. First, the cell loses its function. Second, the necrotic cell's environment gains access to previously hidden surfaces. Necrosis, therefore, is likely to activate resident immune cells. Upon additional production of pro-/anti-inflammatory cytokines, tissue hormones, and lipid peroxides in addition to the unregulated release of subcellular structures, such as broken mitochondria, peroxisomes, endoplasmic reticulum, nuclei etc., specific necrotic signaling pathways may modulate the immune response. Consequently, necroinflammation comes in many different flavors, but, as pointed out in detail, in the nine reviews in this special issue in Cell Death and Differentiation, detailed mechanistic insights are missing and future work is awaited to dig deeper into this clinically important topic. The need to interfere and pharmacologically inhibit regulated necrosis (necroptosis and ferroptosis) is obvious to clinicians working on intensive care units (ICUs) and in the emergency department. Acute myocardial infarction, stroke, acute kidney injury, acute liver failure, every solid organ transplant, pancreatitis, sepsis, and every severe intoxication are only the most prominent devastating diseases to be associated with necroptosis and ferroptosis. Solid cancers clearly are dominated by necrotic areas, the function of which still remains elusive. However, even therapeutic efforts, such as blood transfusion, anticoagulation, antibiotic treatment, and antihypertensive medication, may ultimately result in necrosis in vital tissues. This list is certainly incomplete, but it may highlight the importance to study regulated necrosis and its inflammatory consequences.

Why is there anything new in the concept of necroinflammation [1]? Cell death researchers have discussed immunogenic cell death for over a decade [2]. The critical difference, in our opinion, is in the exclusion of apoptosis as a cause of necroinflammatory diseases. Therefore, necroinflammation contains specificity for non-physiological conditions. In this scenario, it is tempting to speculate about a “specificity for pathophysiology” when necroinflammation is discussed. However, with respect to alternative interpretations of this very young field, it is appreciated that secondary necrosis following caspase-3 activation causes a pyroptosis-like and gasdermin-dependent necrotic cell death that was previously interpreted as secondary necrosis [3]. In addition, caspase-3-mediated effects are by no means limited to apoptosis, but were recently discovered to cleave another gasdermin (GSDME) [4]. These are two examples of caspase-mediated non-pyroptotic necrosis that releases DAMPs. However, necrosis driving activation of caspase-3 may hardly have anything to do with the bulk apoptosis occurring billionfold in vertebrates every day, which again points to some specific pathophysiological setting. Therefore, the hypothesis that there is no physiological role for regulated necrosis still stands. It is along similar lines that RIPK3-ko, MLKL-ko, GSDMD and GSDME-ko mice, and even high concentrations of small-molecule inhibitors of regulated necrosis, such as RIPK1 inhibitors (necrostatins) and inhibitors of specific lipid peroxidation downstream of glutathione depletion (ferrostatins), do not cause any significant spontaneous phenotype or side effects, respectively.

The most conclusive reason for the evolutionary conservation of the necroptosis, the defence against caspase-inhibitor-expressing viruses, is discussed by Nailwal and Chan [5]. This selective advantage comes with the need to remove necrotic debris, e.g. by LC3-associated phagocytosis, as discussed by Martinez and colleagues [6]. Oberst and colleagues summarize the details of the necroptosis machinery and its role in inflammation [7]. Berghe and Hoste add a different perspective in critically reviewing our current concept with a perspective also on other pathways of regulated necrosis [8]. Vince and Frank highlight the crosstalk between apoptosis, necroptosis, and pyroptosis, potentially to be explained by the differences in immunogenicity [9]. Proneth and Conrad focus on the ferroptosis-induced necroinflammation, a hardly investigated research area with paramount clinical importance [10]. Green and colleagues, in this issue, describe the role of necroinflammation in neuronal death and neurodegeneration [11]. The clinical relevance of necroinflammation is the focus of our own contribution to this issue [12], with a special focus on hematopoietic metabolism and disease by Jost and Höckendorf [13].

Modern techniques are essential for further understanding necroinflammation in the near future. Cell death researchers are encouraged to use intravital microscopy to demonstrate the exact moment of cellular demise in mice and/or model organisms. Along similar lines, primary or iPS cell-derived organoids may help to analyze the non-cell-autonomous effects of different necrosis pathways. Clearly, for the research on necroinflammation, we have reached the limits of classical cell culture.

## References

[1] Sarhan M, Land WG, Tonnus W, Hugo CP, Linkermann A (2018). Origin and consequences of necroinflammation. Physiol Rev.

[2] Casares N, Pequignot MO, Tesniere A, Ghiringhelli F, Roux S, Chaput N (2005). Caspase-dependent immunogenicity of doxorubicin-induced tumor cell death. J Exp Med.

[3] Rogers C, Fernandes-Alnemri T, Mayes L, Alnemri D, Cingolani G, Alnemri ES (2017). Cleavage of DFNA5 by caspase-3 during apoptosis mediates progression to secondary necrotic/pyroptotic cell death. Nat Commun.

[4] Wang Y, Gao W, Shi X, Ding J, Liu W, He H (2017). Chemotherapy drugs induce pyroptosis through caspase-3 cleavage of a Gasdermin. Nature.

[5] Nailwal H, Chan FK. Necroptosis in anti-viral inflammation. Cell Death Differ. 2018. 10.1038/s41418-018-0172-x. 10.1038/s41418-018-0172-x PMC6294789 30050058

[6] Kim EH, Wong S-W, Martinez J. Programmed necrosis and disease: we interrupt your regular programming to bring you necroinflammation. Cell Death Differ. 2018. CDD-18-0390R. 10.1038/s41418-018-0179-3 PMC6294794 30349078

[7] Messmer MN, Snyder AG, and Oberst A. Comparing the effects of different cell death programs in tumor progression and immunotherapy. Cell Death Differ. 2018. CDD-18-0691RR. 10.1038/s41418-018-0214-4 PMC6294769 30341424

[8] Berghe TV, Hoste E. Precision medicine 2.0, paving the way for profiling necroinflammation in bio-fluids. Cell Death Differ. 2018. 10.1038/s41418-018-0196-2. 10.1038/s41418-018-0196-2 PMC6294775 30201975

[9] Frank D, Vince JE. Pyroptosis versus necroptosis: similarities, differences and crosstalk. Cell Death Differ. 2018. 10.1038/s41418-018-0173-9. 10.1038/s41418-018-0212-6 PMC6294779 30341423

[10] Proneth B, Conrad M. Ferroptosis and necroinflammation, a yet poorly explored link. Cell Death Differ. 2018. 10.1038/s41418-018-0173-9. 10.1038/s41418-018-0173-9 PMC6294786 30082768

[11] Heckmann BL, Tummers B, Douglas R. Green crashing the computer: apoptosis vs. necroptosis in neuroinflammation. Cell Death Differ. 2018. CDD-18-0702R. 10.1038/s41418-018-0195-3 PMC6294765 30341422

[12] Tonnus W, Gembardt F, Latk M, Parmentier S, Hugo C, Bornstein SR et al. The clinical relevance of necroinflammation—highlighting the importance of acute kidney injury and the adrenal glands. Cell Death Differ. 2018. 10.1038/s41418-018-0193 10.1038/s41418-018-0193-5 PMC6294800 30224638

[13] Jost PJ, Höckendorf U. Necroinflammation emerges as a key regulator of hematopoiesis in health and disease. Cell Death Differ. 2018. 10.1038/s41418-018-0194-4. 10.1038/s41418-018-0194-4 PMC6294770 30242210

